# Recent development of biodegradable synthetic rubbers and bio-based rubbers using sustainable materials from biological sources

**DOI:** 10.1039/d2ra06602e

**Published:** 2022-11-30

**Authors:** Zhen Hern Boon, Yin Yin Teo, Desmond Teck-Chye Ang

**Affiliations:** Department of Chemistry, Universiti Malaya 50603 Kuala Lumpur Malaysia desmond860108@um.edu.my

## Abstract

Rubber is an amorphous hyperelastic polymer which is widely used in this modern era. Natural rubber is considered the ultimate rubber in terms of mechanical performance, but over the years, some limitations and challenges in natural rubber cultivation that could result in serious shortages in the supply chain had been identified. Since then, the search for alternatives including new natural and synthetic rubbers has been rather intense. The initiative to explore new sources of natural rubber which started during the 1940s has been reignited recently due to the increasing demand for natural rubber. The commercialization of natural rubber from the *Parthenium argentatum* and *Taraxacum kok-saghyz* species, with the cooperation from rubber product manufacturing companies, has somewhat improved the sustainability of the natural rubber supply chain. Meanwhile, the high demand for synthetic rubber drastically increases the rate of depletion of fossil fuels and amplifies the adverse environmental effect of overexploitation of fossil fuels. Moreover, rubber and plastic products disposal have been a major issue for many decades, causing environmental pollution and the expansion of landfills. Sustainable synthetic rubber products could be realized through the incorporation of materials from biological sources. They are renewable, low cost, and most importantly, biodegradable in nature. In this review, brief introduction to natural and synthetic rubbers, challenges in the rubber industry, alternatives to conventional natural rubber, and recent advances in biodegradable and/or bio-based synthetic rubbers are discussed. The effect of incorporating various types of biologically sourced materials in the synthetic rubbers are also elaborated in detail.

## Abstract 2

Recent progress to improve the sustainability and biodegradability of synthetic rubber by incorporating raw materials from biological sources.

## Graphical abstract

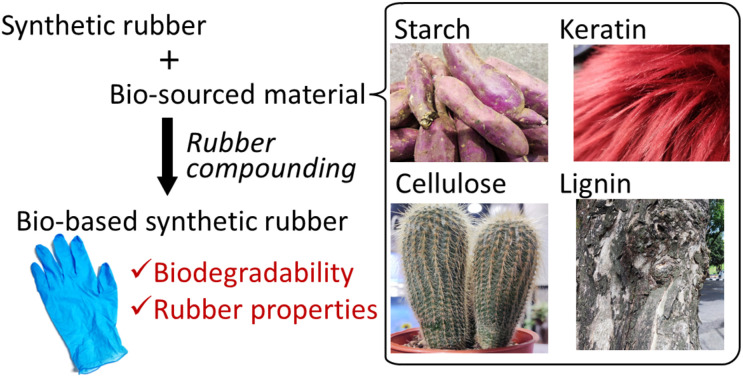

## 1.0 Introduction to rubber and the challenges behind natural rubber industry

Rubber is a class of polymer categorized as elastomer, an amorphous hyperelastic material having sub-ambient glass transition temperature. Rubber can be divided into 2 types, natural rubber and synthetic rubber, depending on the origin. Natural rubber was first used back in prehistoric times (Olmec and Mayans) as rubber balls in sports, which were important religious and political events during that time.^1–4^ The industrialization of natural rubber began following the discovery of the vulcanization process by Charles Goodyear in 1839. The vulcanization process managed to solve the low-temperature brittleness and high-temperature softening of natural rubber,^5–7^ and subsequently revolutionizes the rubber industry and increases the demand for natural rubber drastically. This induced the Hevea seedling smuggling incident in 1876 to hinder the monopolization of the price of natural rubber as Brazil was the sole natural rubber exporter during that time. The seeds were then replanted in Malaysia, and this sparked the dawn of the natural rubber industry in Southeast Asia as the major natural rubber suppliers even until today. Currently, countries in Southeast Asia inclusive of Thailand, Malaysia, and Indonesia contributed to over 90% of global natural rubber supplies.^7,8^ The production, harvesting, and processing of natural rubber had matured over these years, bolstered by the suitable climate and growth conditions, cheap labour costs, and the high acreage of land available for rubber plantations in developing countries. However, several limitations and challenges associated with the natural rubber industry had been identified over time and these necessitate the search for new or alternative sources of rubber material, both natural and synthetic.

Current *H. brasiliensis* plantations in Southeast Asia are of the same origin as the seeds brought in during the 19th century. *H. brasiliensis* trees have very little genetic variability^5,7,9^ and have limited resistance to diseases such as South American Leaf Blight (SALB) or white rot disease.^7,9,10^ It was reported that infection in one of the trees may wipe out the whole plantation in no time and causes a shortage in natural rubber latex supply for future years. Besides the vulnerability to infection, the supply of natural rubber is further constrained by the strict conditions needed for optimal growth of the trees, which include the need for tropical climate that provides warm environment with abundant rainfall and sunlight. This excludes non-tropical countries from cultivating *H. brasiliensis* and effectively limit the global supply of natural rubber. Moreover, some of the tropical countries such as Malaysia have gradually replaced rubber plantations with palm oil plantations which are more profitable due to the increased biofuel demand,^10–12^ and this has further exacerbated the natural rubber supply chain problem.

The applications of natural rubber cover a wide range of products, including examination and surgical gloves. The use of natural rubber gloves, also known as latex gloves first increased during the HIV epidemic in the 1980s and has grown exponentially with the recent COVID-19 pandemic since 2020.^13–15^ Latex allergy incidents have also been increasing dramatically, with around 6% of the population exhibiting latex sensitization.^15^ Latex allergy caused by the proteins in natural rubber latex could result in varying reactions ranging from moderate symptoms such as itchy skin and hives, to life-threatening symptoms such as anaphylaxis, difficulty in breathing, and/or severe vomiting^16^ through direct contact or particle inhalation by sensitized personnel. There has been numerous studies and initiatives to address the latex allergy issue caused by the natural rubber gloves. The complete deproteinization of latex had shown a negative effect on the mechanical property of the rubber, as the non-rubber components, including the allergy-associated proteins were found to be responsible for the formation of island-nanomatrix structure that strengthened the rubber and stabilised the latex.^17,18^ Other methods developed by the glove manufacturers such as modification of leaching protocol to leach out the soluble proteins near glove surface and/or applying polymer coatings such as hydrogel or silicone polymer on the gloves' surface to prevent direct contact with the wearer have shown varying degrees of effectiveness.^19,20^ These additional steps are however responsible for the increase in the production cost and the price of latex gloves.^17,21^

Unfortunately, after more than a century of industrialization, the natural rubber industry still relies on only a single species of natural rubber-producing crop as the predominant source of natural rubber. Given the challenges and constraints to increase the existing natural rubber supply, coupled with the high demand for the natural rubber in various industries,^22–24^ there is an urgent need to exploit other rubber materials as potential substituents to the current Hevea rubber. The following section of this review details the alternative sources of natural rubber, as well as recent progress in synthetic rubbers.

### 1.1 Alternative natural rubbers

Throughout the mid-20th century, there have been many attempts to search for alternatives to Hevea rubber. The main reason behind it was the cut-off of natural rubber supply from Southeast Asia due to the Japanese colonization during World War II.^25^ More than 2500 types of plants were found to be capable of producing latex, but only two, *Parthenium argentatum* and *Taraxacum kok-saghyz* were shortlisted as alternative sources of natural rubber with promising potential to be cultivated commercially.^25–27^

*Parthenium argentatum*, also known as Guayule is a shrub native to the Chihuahuan desert of Texas and northern Mexico, and it is used as the rubber source for the local population there.^11,25^ The discovery of Guayule rubber was seen as a promising alternative source for its hypoallergic characteristic due to the lack of proteins associated with the latex allergy.^9,11,25,28,29^ However, Guayule rubber was found to have inferior quality compared to Hevea rubber, and this includes the presence of high resin content and relatively low molecular weight rubber component.^9^ Before Guayule rubber can serve as a substituent to Hevea rubber, extensive research and breeding techniques are required to address the abovementioned limitations. Initial research focused on the yield had produced promising results, with significantly higher yield and high-quality guayule rubber obtained. Unfortunately, the yield was rather unstable, and the higher production cost involved compared to producing Hevea rubber has halted the research. It is noteworthy that the price of Hevea rubber was relatively cheap when it became widely available after the World War II period. Nevertheless, Guayule remains as one of the attractive and potential alternatives to Hevea rubber, with its high rubber yield and low water requirement that allows it to be cultivated in non-tropical regions.^9,30^ Guayule however does not tolerate prolonged freezing temperature which covers the USA and much of Europe during the winter, as this will freeze the latex and greatly inhibits Guayule's growth rate.^29,31,32^ At present, although not many, there are isolated reports on the use of Guayule rubber in product innovation and these include Guayule/Neoprene blend wetsuits and Guayule-derived tires commercialized by Yulex Corporation and Bridgestone, respectively.

**Fig. 1:**
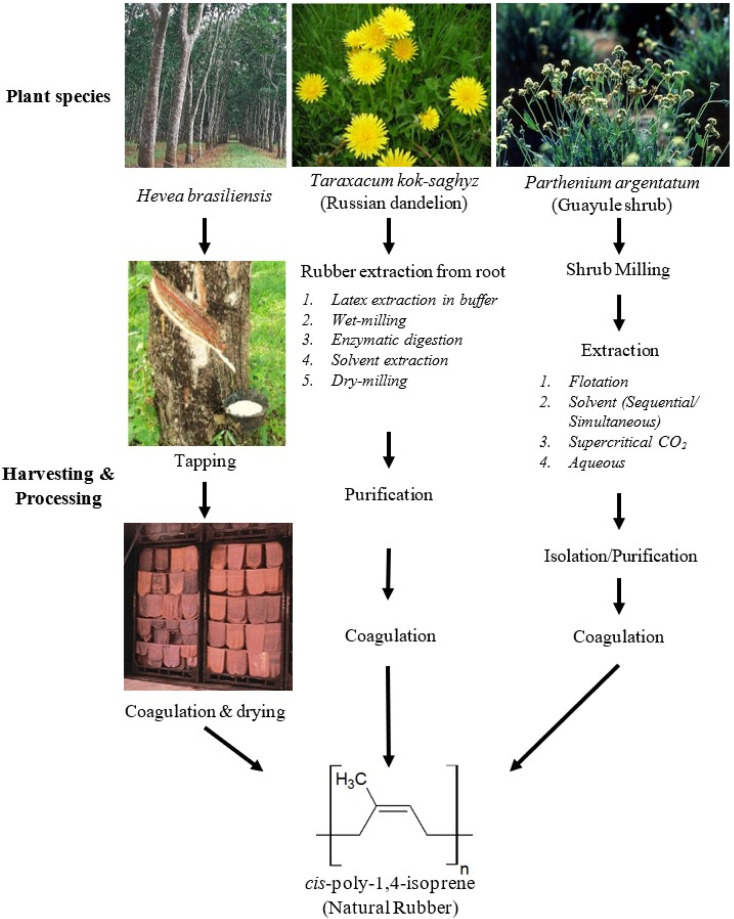
Natural rubber producing plant species and their general industrial harvesting process.^33–39^

*Taraxacum kok-saghyz*, or Russian dandelion, originates from Kazakhstan and was discovered in the USSR in the effort to search for domestic sources of natural rubber in 1931. It is an annual crop that has a short life cycle of around 6 to 8 months,^9^ which is significantly shorter compared to *Parthenium argentatum* (2 years) and *Hevea brasiliensis* (minimum 10 years growth required before producing rubber). The short life cycle of Russian dandelion plant eases the research and cultivation study, making it one of the prime candidates to substitute Hevea rubber. Fig. 1 summarizes the processes involved in harvesting natural rubber from the three plant species mentioned above. The cultivation and harvest of Russian dandelion are labour-intensive, has a low yield per hectare, the seedlings are small, easily outcompeted by weeds, and crossbreeding results in contamination between different species of dandelions.^11,12^ This makes vigorous tilling required for a Russian dandelion plantation to be set up.^9^ The compositional analysis rubber reveals high-quality of Russian dandelion rubber comparable to that from *Hevea brasiliensis* by having very high molecular weight. The rubber is as resilient as the Hevea rubber and is better than Guayule rubber. However, it has more associated protein than the Hevea's, making the latex more sensitizing and increases the chances of allergy reaction with symptoms likely to be more serious. With these traits, Russian dandelion rubber is more suitable for non-medical applications or for heavy usage such as tires. Russian dandelion rubber made up 30% of the rubber supply in USSR during World War 2. However, similar to what had happened with Guayule, most if not all research on the cultivation of Russian dandelion was abandoned when the supply of Hevea rubber resumed after World War II.

**Fig. 2:**
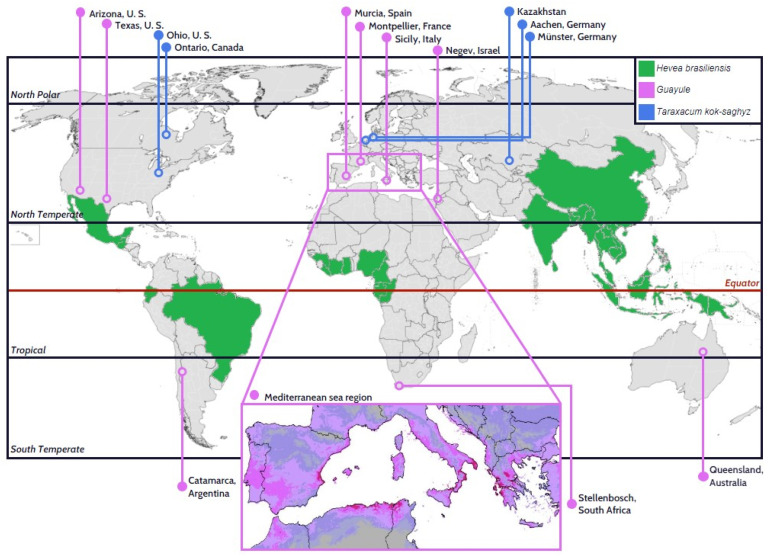
Current global distribution of natural rubber plantations (Hevea = commercial scale, Guayule & Russian dandelion = commercial, research and trial), and inset showing feasibility test site of guayule plantation in Mediterranean region.^44–47^

The globally rapid expanding economies, especially in China, who is the biggest consumer of natural rubber, have seen a rapid yearly increase in demands for natural rubber over the past decades.^23^ The saturation of rubber plantations in the “rubber-belt” area and the irreplaceability of natural rubber by the synthetic alternatives had reignited the world's interest to search for alternative sources of natural rubber to cope with the increasing demand for the rubber. One of the solutions was to source for natural rubber from different rubber producing plant species that can be cultivated and expanded to non-tropical regions. For example, Yulex, a corporation that commercialized Guayule-based products focusing on apparel such as wetsuits, swimming and diving suits, and footwear, has its own Guayule plantation and processing facilities.^40^ Bridgestone has Guayule research facilities in Arizona since 2012 and has launched racing tires made of guayule-derived rubber.^41^ Kultevat and KeyGene biotech companies collaborated on the research in Russian dandelion rubber towards the goal of commercialization.^42^ Continental launched truck tires made from Russian dandelion-derived rubber in 2016.^43^ Although these initiatives provided some degree of sustainability to the supply of natural rubber, the yield and application of the alternative natural rubbers is considerably low compared to the well-established Hevea rubber. The global distribution of natural rubber plantations, inclusive of Hevea, Guayule and Russian dandelion are shown in Fig. 2.

## 2.0 Synthetic rubber and the need for sustainability

**Fig. 3:**
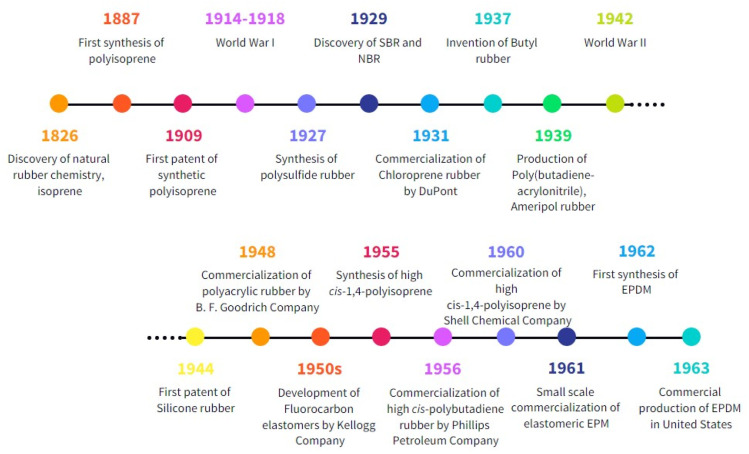
Timeline of the development of different synthetic rubbers.^48^

**Fig. 4:**
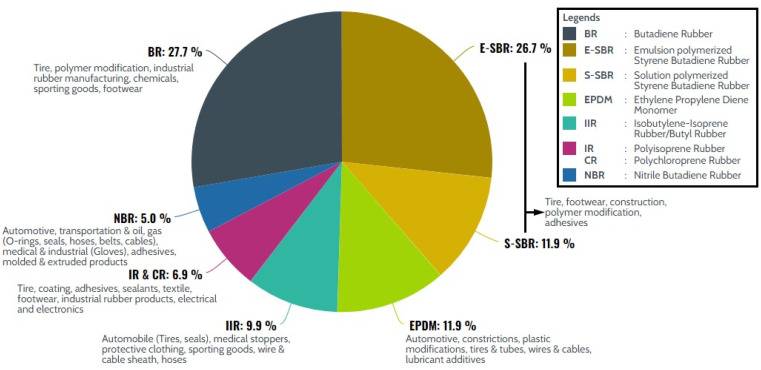
Demand for synthetic rubber in 2020 according to rubber types, and their respective applications.^49–57^

Synthetic rubber refers to any artificial rubber synthesized from monomers derived from petroleum by-products or those of natural origin. The former however made-up bulk of the synthetic rubbers that are in use today. Since the first successful synthesis of synthetic polyisoprene in 1887, there were many variations of synthetic rubbers developed throughout history. The brief timeline of the development of synthetic rubbers is shown in Fig. 3. Until today, there are more than 20 types of synthetic rubbers that are widely available in the market. Fig. 4 shows the data on demand and distribution of synthetic rubber by application in the year 2020, while a summary of the industrial production methods and properties of some commonly used synthetic rubbers is given in Table 1. Synthetic rubbers not only share many similar applications as natural rubber, but also provide an extended range of applications. The primary cause is that synthetic rubber is made using a wide range of monomers, resulting in a variety of rubber properties to be engineered, as opposed to natural rubber, which typically has just one chemical structure. For example, NBR has excellent oil resistance contributed by the polar nitrile group from acrylonitrile moiety in the rubber. The mechanical properties, oil, and chemical resistance of the rubber could be altered by manipulating the ratio of butadiene to acrylonitrile repeating units. Over the decades, the oils and gas industry has optimized the extraction and processing of crude oil and natural gas, resulting in the availability of cost-effective raw materials for synthetic rubbers. This is presumably one of the reasons to most of the synthetic rubber formulations and processing technologies used in the rubber industries today are still largely similar to those that were developed in the early days of synthetic rubber discovery.

**Table 1:** General description, properties, and applications of conventional synthetic rubbers

Rubber	Monomers	Industrial synthesis method	Properties	Applications	Ref.
Styrene butadiene rubber (SBR)	Styrene	Hot emulsion polymerization	More branched, lower molecular weight chain	Lightweight vehicle tire (passenger car, radical car, motorcycle thread), electrical insulation, hoses, seals, haul-off pads, shoe soles	49–52
	Butadiene		Higher *T*_g_ than cold & S-SBR		
			Good dimensional stability		
			Good extrusion characteristics		
		Cold emulsion polymerization	Better abrasion resistance, tensile strength than hot e-SBR		50 and 51
		Anionic solution polymerization	High molecular weight linear chain		50 and 51
			Narrow molecular weight distribution		
			Excellent abrasion and fatigue resistance, mechanical properties		
			Better tire tread traction properties		
Chloroprene (Neoprene)	2-Chlorobutadiene	Emulsion polymerization	High oil and solvent resistance	Gloves, shock absorber seals, electrical insulation, asphalt, wetsuits, bearing pads	51 and 53
			Good thermal, weather, ozone and aging resistance		
			High strength and stiffness resulted from stress-crystallization		
Butyl rubber (IIR)	Isobutylene	Cationic slurry polymerization	Excellent water, air and gas permeability resistance	Stopper, gas masks, sealant, gasket, hoses, o-rings, chewing gum base, waterproof liners	51 and 54
	Isoprene		Good low temperature flexibility		
			Low compatibility with other polymers, improved by halogenation (CIIR & BIIR)		
Nitrile rubber (NBR)	Acrylonitrile	Hot emulsion polymerization	Excellent oil and solvent resistance	Engine hoses, seals, gloves, oil and hydraulic seals, o-rings, waterproof fabrics, adhesives, pigment binder	51
	Butadiene		High green strength, low flexibility		
		Cold emulsion polymerization	Less branching chain than hot polymerized		51
			Better processability than hot-NBR		
Ethylene propylene diene rubber (EPDM)	Ethylene	Solution polymerization	Wide temperature range of application	Roofings, radiator hoses, door seals, gaskets, electrical connectors & insulators, water tank liners	51, 55 and 56
	Propylene		Good weather, UV, ozone and aging resistance		
	Dienes (1,4-hexadiene *etc.*)		Good resistance to polar fluids, low non-polar solvent resistance		
			Low compatibility with other polymers (saturation)		

Since the 1960s, rapid technology and industrial development had raised global concerns about the detrimental impact on the environment and subsequently sparked the concept of sustainable development later in the 1980s. The primary concept of sustainable development is to ensure that the present needs that could be achieved now should not be compromising the future generations' ability to meet their needs. One of the goals of the United Nation's Sustainable Development Goals (SDGs), “Responsible Consumption and Production”, targeted the efficient use of natural resources can be alternatively defined as the resources that were used in any product development should be able to be replaced naturally.^58^ Besides, the 6th principle of Green Chemistry, a guidance developed by the United States Environmental Protection Agency for the design of chemical processes of products emphasized the use of renewable raw materials rather than the depletion of the economically available ones.^59,60^ Both of them targeted sustainability at its source. Unfortunately, this is not the case in the synthetic rubber industry as the raw materials are mostly derived from non-renewable fossil fuels. With the paradigm shift towards sustainable development and green chemistry, the search for sustainable elastomers has been intense in the last 10 years, and the desperation also arises from the ecological and environmental effects of the overexploitation of fossil fuels that had been evidently amplified.

One of the approaches to foster sustainability was through the incorporation of conventional monomers obtained from biomass sources. Biomass is organic material from biological beings. Some of the common synthetic rubber monomers could be obtained from reprocessing biomass fermentation products. For instance, carbohydrate of biomass waste such as crops and wood is hydrolyzed sequentially by acids and enzymes to produce sugar such as sucrose, which are then fermented to yield bioethanol.^61^ In the case of EPDM, ethylene and propylene can be obtained from the bioethanol through dehydration and addition reaction with suitable catalysts.^62,63^ In recent years, some rubber manufacturing companies incorporated these bio-based monomers in their rubber productions. Companies such as Bridgestone, Goodyear, and Michelin incorporated bio-sourced isoprene into their tire products in the 2010s.^64^ According to a statistical report, the usage of biomass-derived chemicals was at 2.6% of total usage in year 2020.^65^ Although the number is increasing steadily in the past 10 years, it is nowhere close to cope with the rate of depletion of fossil fuels anytime soon. Refined protocols that could increase the production efficiency and cost-effectiveness of the bio-sourced monomers, as well as cooperation from large polymer manufacturing companies may be necessary to ensure the viability of this initiative.

Another approach to producing sustainable rubber was achieved with the use of raw materials from natural sources in rubber product development. This includes materials in the form of monomers, as well as compounding materials such as fillers or additives. Incorporation of these non-conventional materials produced novel rubber materials or composites with properties that are of interest and worth investigating. Over the past decade, there are many studies and reports on the use of naturally produced raw materials in the synthetic rubber development. The studies are largely divided into two groups with different focuses; one of them focuses on improving the performance of the rubber (mechanical, thermal, and chemical resistant properties) while ensuring its sustainability, and another prioritizes the biodegradability of the novel synthetic rubber. Naturally occurring compounds are known for their ability to be degraded naturally, hence it is reasonable to expect synthetic rubber comprising of such compounds to exhibit a certain extent of biodegradability. Unfortunately, the difference in priorities in the studies are distinct, with many of the studies that investigate the improvement in the performances of the rubber compounded with natural raw materials did not report the biodegradability of the rubber. Due to this, the following review section is divided into two segments, beginning with the review on the recent advances in the use of natural materials to enhance the biodegradability of synthetic rubbers in Section 3.0, and the following Section 4.0 focuses on the sustainability and improvement of the properties of synthetic rubbers using biologically sourced materials.

## 3.0 Recent progress in enhancing biodegradation of synthetic rubbers

The fact that polymeric materials generally do not decompose easily applies to synthetic rubber as well and they take a long time to be degraded in nature. The synthetic origin of the rubbers is one of the contributing factors to their limited biodegradability, with most of the raw materials used in the production of rubber being derived from fossil fuels. The availability of a wide range of types of monomers that can be used to synthesize rubber has resulted in a vast variety of synthetic rubbers with unique chemical compositions. Besides, various polymerization techniques have been adopted and resulted in rubber with different molecular weight, structure, configuration, and the use of various petroleum-based additives in the rubber compounding process also adds up to the complexation when it comes to the biodegradation of synthetic rubber.^66^ Many other factors are also known to affect biodegradation activity such as the degradation environment which includes the types of microbial species present, temperature, pH, and aerobic or anaerobic surroundings. The disposal of rubber products after their end-of-life cycle and poor waste disposal management are among the problems faced globally.^66–68^ The high demand and usage volume for rubber products created a serious problem, as the rate of biodegradation of synthetic rubbers is extremely low, and they persist in landfills or nature for almost indefinitely after being discarded, causing environmental pollution and increase in landfill sizes.^69^

In general, biodegradation is defined as the process to breakdown bigger molecules into smaller products by the activity of microorganisms. Naturally occurring materials tend to produce smaller environmentally friendly organic by-products through biodegradation,^66^ and ultimately the products can be further digested to produce CO_2_, H_2_O and/or CH_4_. The mechanism of biodegradation involves the secretion of enzymes by the microbes to initiate the breakdown of the material, and it is essential for the microbes to be able to utilize carbon sources from the material substrate. In general, microbes are not able to consume or digest synthetic rubbers, hence the low microbial activity and non-biodegradability of those rubbers.^67^ This situation is worsened with the use of non-biodegradable additives during the rubber compounding phase. In conventional rubber production, the rubber compounding phase is an inevitable process to render the rubber practically useful for many applications. The most significant part of rubber compounding is the vulcanization process to create crosslinking between rubber chains for the improvement of physical and mechanical properties. Fillers and other additives are often incorporated during this phase; for example, reinforcement fillers for further improving the properties and dyes for changing the appearance of the product. In recent years, there are many reports on the use of renewable materials as additives or as blending components for synthetic rubber, and the incorporation of such materials has resulted with improvement in the biodegradability of the products.

### 3.1 Starch

There is an increase in the number of research that focuses on the incorporation of natural fillers in synthetic rubber, and the purpose of the additive is to not only emulate the conventional role of fillers but to serve as nutrients for the microbes during the biodegradation process as well. One example of such fillers is starch, a type of polymer categorized as carbohydrates and consists of glucose repeating units. Starch is widely available in almost all plants as the product of photosynthesis, and as the energy storage unit in plants. It has been utilized in the development of bioplastics because of its high availability and renewability, low cost, and biodegradability.

Rubber blending with starch is another approach to incorporating biodegradability in synthetic rubber. The blend composite of cassava starch (CS)/carboxylated styrene butadiene rubber (XSBR) was reported by Riyajan *et al.* in 2018.^70^ Potassium persulfate initiator was included in the blend to induce the grafting of CS to XSBR through the carboxyl group, preventing the agglomeration of starch particles due to the difference in hydrophilicity between the starch and the rubber. In general, the increase in the ratio of CS in the blend resulted in an increase in the rate of soil biodegradation, TS, and water swelling ratio. The highest mass loss of 80% in 1 month was reported with CS/XSBR ratio of 1 : 1. The high TS of 25 MPa was comparable to the conventional synthetic rubber, but EB of the film was considerably low at about 100% only, resulting in a strong but relatively brittle material. The TS, EB and biodegradability could be tuned with different ratio of cassava starch in the blend and a balance in these properties are necessary to produce a sustainable and biodegradable rubber blend with high mechanical performance.

In a separate study, glycerol was used as a plasticizer in CS/XSBR blend to largely improve the EB, and cellulose fibre fillers were introduced to control the moisture content. Although improvement in the mechanical property was observed, the biodegradability of the composite slightly decreased, from full disintegration to 82.6% weight loss within 30 days.^71,72^

The presence of rich hydroxyl groups in starch molecules promote agglomeration as a result from the strong hydrogen bonding among starch molecules.^73^ Modification of starch molecules is often required for good dispersion and subsequently good resultant properties of the rubber.^74^ In 2019, Daud *et al.* incorporated acid hydrolyzed sago starch (AHSS) as filler in carboxylated nitrile rubber (XNBR), showing improved biodegradability of the composite.^75^ The AHSS-filled XNBR showed 22% mass loss in the soil burial test after 3 weeks. The percentage of mass loss in both samples of AHSS-filled or native sago starch-filled XNBR were much higher than the unfilled XNBR (10% mass loss) at 22% and 17%, respectively. However, AHSS is not a good reinforcing filler in XNBR as the mechanical properties such as tensile strength (TS) and elongation at break (EB) of the filled rubber are inferior to the unfilled control. The demotion of mechanical properties is due to the poor interfacial interaction that resulted from the high hydrophilicity difference between hydrophilic starch molecules and the hydrophobic rubber matrix.

Amino-functional starch (ANS) was synthesized by Misman *et al.* by graft copolymerization of starch molecules with acrylonitrile, a monomer containing the same nitrile functional group as XNBR.^76^ While the introduction of 10 phr ANS filler in XNBR did create starch interstitial region that reduced the crosslink density like in the case of AHSS, the EB of the rubber improved, and TS was insignificantly affected. This was attributed to the glucose ring opening *via* amino functionalization where the hydrogen bonding between the two sides of the ring-opened glucose units provides reversible points to dissipate external stress in the starch chain before total structure failure occurs. ANS-filled XNBR also has significantly improved biodegradability, with 25% mass loss over 8 weeks of soil burial test. The formation of microbial coaggregation was also observed through a scanning electron microscope (SEM) in the soil burial test samples.

Starch-*graft*-poly(methyl methacrylate) produced through emulsion polymerization between natural corn starch powder and methyl methacrylate as a filler could improve the mechanical properties of SBR through the physical entanglement between SBR matrix and starch grafted PMMA.^77^ The soft, high strength and bendable material are suggested for applications in tight packaging such as fruit wrapping. The rate of biodegradation increases tremendously at a high filler content of >30 phr but the mechanical strength deteriorates worse than the unfilled SBR due to agglomeration as a consequence of high filler–filler interaction overrides the mechanical reinforcement resulting from the physical entanglement. At optimum 30 phr filler content, 2% mass loss of samples could be obtained after 90 days of soil burial.

### 3.2 Cellulose

Cellulose is a polysaccharide composed of a linear chain of β-1,4 linked d-glucose units and is one of the most abundant organic polymers on earth. In 2019, Taib *et al.* modified the properties of nanocellulose using gallic acid as antioxidant and subsequently introduced it into synthetic rubber.^78^ The antioxidant nanocellulose (Aox-NCC) acts as a reinforcement filler and crosslinking agent when incorporated into NBR. The optimum amount of Aox-NCC reported was 3 phr, providing the maximum mechanical strength improvement through the increase in crosslink density, before the decrease in efficiency of reinforcement due to the agglomeration of Aox-NCC particles. The resulting fillers have a negligible mass loss during the soil burial degradation test within 6 months in both filled samples and unfilled control. According to the author, the antioxidant moieties act as radical removers during oxo-biodegradation, slowing down the overall biodegradation process and further investigation is required to conclude the biodegradability of Aox-NCC. Cellulose occurs abundantly in plants as an important constituent of cell walls, hence cellulose fibres can be extracted from many sources, for example, *Opuntia Cactaceae* cactus trunk, in the form of the mesh-like fibrous network.^79^ A high extent of degradation approaching complete disintegration can be achieved within 7 months with the incubation of the cellulose fibre network (9 wt%) filled SBR composite in organic soil. Besides plant cellulose sources, bacterial cellulose is also frequently used as filler for synthetic rubber due to its high purity, crystallinity and unique open network structure leading to a large surface area.^80^ Moreover, hydrolysis of bacterial cellulose resulted in bacterial cellulose whiskers, forming a rigid 3D filler network through hydrogen bonding between whiskers. Water-induced stimuli-responsive SBR reinforced with bacterial cellulose whiskers was reported by Chen *et al.* They suggested the rupture of the said 3D network occurs during the presence of other competitive hydrogen bonding agents, such as water. This results in a stimuli-responsive composite for the applications such as sensors.^81^

### 3.3 Keratin

Keratin is also one of the natural raw materials that have received attention from the rubber industry. Keratin refers to one of the families under scleroproteins, a type of structural fibrous protein. Keratin consists of peptide chains, forming different functional components in biological organisms for the role of support and protection, such as the epithelial tissues which include skin and nails. Keratin for use in synthetic rubber is usually extracted from the tannery and poultry waste of vertebrate sources such as sheep wool and bird feathers. Even though the occurrence of keratin in these wastes is not as high as collagen, the extraction of keratin from these wastes involves relatively low cost and could reduce the environmental damage and pollution compared to the fossil fuel-based materials. The use of keratin as a biodegradable component in XNBR has been reported by Prochoń *et al.*^82^ XNBR filled with cattle hair keratin and its acid hydrolysate showed improved crosslink density and higher oil resistance as diesel fuel and leaded petrol compared to unfilled XNBR. Cattle hair keratin showed better mechanical performance reinforcement than hydrolysate due to the higher specific surface area, smaller particle size and the high molecular weight, while in keratin hydrolysate, the large particle size from the formation of aggregates by the small molecular weight scissored chains during acid hydrolysis resulted in poor filler dispersion and lower reinforcement effect. The better filler dispersion is also seen to affect the biodegradability, with the keratin-filled XNBR exhibiting a greater reduction in TS (−30%) and crosslink density after 30 days of burial test in universal soil, indicating the higher decomposition activity by microbes. Keratin hydrolysate from bird feathers (bird feather hydrolysate, BFH) reinforced XNBR and NBR could achieve a faster cure rate, and high strength reinforcement but resulted in lower flexibility composites. However, the process of bird feather hydrolysis should not use highly concentrated alkali as it could further hydrolyse keratin to release free amine and carboxylate ions, resulting in poor water uptake capability and biodegradability of the composite.^83^

Keratin hydrolysate could also be obtained through enzymatic hydrolysis. The activity of enzymatic hydrolysate of keratin was investigated in sulphur cured system with different additional fillers, poly(vinyl alcohol), (PVAL) and montmorillonite (MMT) clay by Prochoń *et al.* in the year 2015 and 2020, respectively.^84,85^ Generally, the MMT-filled samples have higher TS, while EB and hardness are comparable to PVAL-filled samples at the same amount of keratin fillers. At 10 phr keratin hydrolysate, 3.2% and 5.2% in TS reinforcement were observed in PVAL-filled and MMT-filled samples, respectively. As for the biodegradation test, both samples appeared discoloured, blotchy and uneven surfaces after being buried in soil for 1 month. The discolouration observed through a spectrophotometer was attributed to the biodegradation of the biopolymer part as the colour protection properties were lost. In the MMT-filled XNBR with 5 phr keratin hydrolysate sample, a 50% decrease in TS after 3 months of soil burial was reported. Compared to the negligible change in the TS of the unfilled sample, the reported drop in the filled sample was considerably large, suggesting the use of enzymatic keratin hydrolysate as filler in XNBR could be a very promising approach to produce biodegradable synthetic rubber without compromising the mechanical performance. The effect of inorganic particle-keratin filler blending in NBR was investigated by Prochoń *et al.*^86^ The blending of keratin with zinc oxide before the compounding phase decreased the particle size of the filler and improved the water uptake capability and biodegradability. There was a 13.6% increase in TS loss compared to control in 30 days of controlled soil incubation.

### 3.4 Lignin and collagen

The combination of lignin fibrils and graphene oxide (GO) fillers highly improved thermal stability of SBR, making it suitable for applications such as green tires.^87^ The incorporation of lignin fibrils filler further improved TS and EB of SBR compared with sole GO filler. The optimum amount of lignin fibrils was reported at 2 wt% for SBR which was pre-compounded with 5 wt% of GO, and a high mass loss of approximately 50% in 50 days of biodegradation was achieved by such composite while benefiting from the maximum mechanical reinforcement. Composite with higher content of lignin fibrils, at 3 wt% further increased the biodegradability but EB was largely reduced to a value slightly lower than GO or pure SBR. In general, altering the amount of biodegradable components in the composite is a simple way to tailor their properties according to the intended application of the biodegradable synthetic rubbers. GO filler used together with elastin collagen was also reported recently, with SBR composite sample containing 1.5 wt% collagen in 5 wt% GO exhibited 19% and 24% increase in TS and EB, respectively.^88^ The biodegradability of the SBR/GO/collagen composite increased due to the synergistic effect of GO's naturally bonded carbon structure and the presence of biodegradable collagen.

### 3.5 Other sources of natural raw materials and their combinations

Recently, the use of coconut shell powder, CSP as a filler for SBR was reported. CSP contains a high amount of fibre consisting of cellulose, lignin and pentosans. The compatibility of CSP with rubber matrix was improved through silane modification and PMMA grafting.^89,90^ In general, the biodegradability of the rubber decreased with the increase in the extent of modifications which include the percentage of grafting and amount of silane-modification. The extent of biodegradation of the SBR was less than 5 wt% within 6 months. This implies that the weight loss recorded could be attributed to the biodegradation of the CSP portion in the rubber. PMMA-*g*-CSP filled SBR composite exhibit higher thermal stability due to lower crystallinity structure of filler, and improved mechanical strength, like the case of PMMA-*g*-starch mentioned above that resulted from the increased filler–matrix interaction and formation of physical entanglement.

A tough polyester comprised of peroxide-crosslinked poly(1,4-butanediol/1,3-propanediol/sebacate/itaconate/succinate) has superior mechanical properties after vulcanization and exhibited good compatibility with inorganic fillers.^91^ However, it did not undergo degradation after 3 months of natural soil burial test even with the use of monomers of natural sources such as succinic, itaconic and sebacic acid. This is due to the combined effect of nanoparticle volume effect, high strength of filler–matrix interaction and the carbon–carbon crosslinks between the polymer chains. On the other hand, linear isosorbide-based polyester with fatty acid dimer undergoes 95.1% degradation in 45 days in a controlled soil compost biodegradation setup according to standard method EN 13432.^92^ Increasing the amount of fatty acid dimer in the polyester however resulted in a lower degradation rate due to the increase in hydrophobicity and lower amount of degradation susceptible-ester bonds.

The degradability of synthetic rubber in aqueous medium is also an important consideration as the surface of the earth is covered by almost 70% of water. Disposal of synthetic rubber products could end up in aqueous environments such as rivers, lakes and seas due the unethical disposal or accidental leakage from landfills. The degradation of poly(dimethylsiloxane) (PDMS) which is also known as silicone rubber and characterized by the presence of siloxane (Si–O) bonds in the polymer backbone, in both aqueous and soil media has been reported by Ceseracciu *et al.* in 2015 and Tran *et al.* in 2017, respectively.^93,94^ Although PDMS has excellent thermal stability and can be used for a wide range of operating temperatures, the rubber was found to be degradable in the soil environment through the hydrolysis of siloxane bonds.^95^ The product of siloxane bond hydrolysis can then be further degraded by radicals and microbes into silica, carbon dioxide and water. The degradation products of PDMS are usually environmentally friendly and PDMS is also non-toxic and biocompatible.^95^

The degradation properties of acetoxy-cured PDMS in Mediterranean seawater had been investigated and reported recently while incorporating different natural fillers, *i.e.*, unmodified corn starch granules and micronized cocoa shell waste (CSW).^93,94^ In both cases, the filled PDMS have higher biological oxygen demand (BOD) when immersed in Mediterranean seawater, and oxygen consumption increased with higher filler loading. PDMS filled with 70% corn starch experienced 5% volume degradation in 60 days, and the PDMS–starch composite is estimated to be fully degraded in seawater in 3–6 years depending on the starch content. The formation of a porous structure with high CSW content improved water permeation into the PDMS network and this contributed to the improved biodegradability of the material. However, from the EDX analysis, no significant amount of elemental silicon was found in the seawater after the test period of 1 month, implying the initial degradation only proceeded in the CSW. High elastic recovery rates and stress relaxation properties of corn starch-filled PDMS suggested that the composite could be used as mechanical energy dampeners because of the low strain energy dissipation factor. Besides, CSW introduced radical scavenging activity into PDMS due to the presence of a high volume of flavonoid and polyphenol in cocoa, which could be useful in active food packaging, cosmetics or biomedical devices involving drug release.

A similar antioxidant property with additional gradual antioxidant release in starch granules–PDMS was achieved with the use of red beetroot powder (RBP) as fillers.^96^ With the increase in RBP content, improvement in TS without adverse effect on the EB was obtained, and this suggests a better performance composite than those filled solely by starch. The rate and amount of antioxidant release, *i.e.*, the antioxidant activity was controllable with the amount of starch granule filler. However, the encapsulation of starch granules in the PDMS network rendered them protected from hydrolytic degradation when immersed in water. Thus, the action of antioxidant betanin release from RBP into the water was through diffusion in the PDMS network. The rate of degradation of synthetic rubber products is affected by the aqueous environment. But in general, degradation of polymeric materials in an aqueous medium is usually slower than in a soil environment due to a lack of suitable degradation conditions for microbial degradation actions.^97^ Hydrolytic/aqueous degradation tests of elastomers sometimes incorporate different conditions, especially the pH of the aqueous medium and one of the reasons for this is due to the differences in pH in water sources around the globe. However, for elastomeric products containing hydrolysable bonds such as polyurethane and polyester, recovery of monomers through acidic, alkaline or chemical degradation could be more prominent as recyclability of resources also contributes to sustainability.^98,99^

**Fig. 5:**
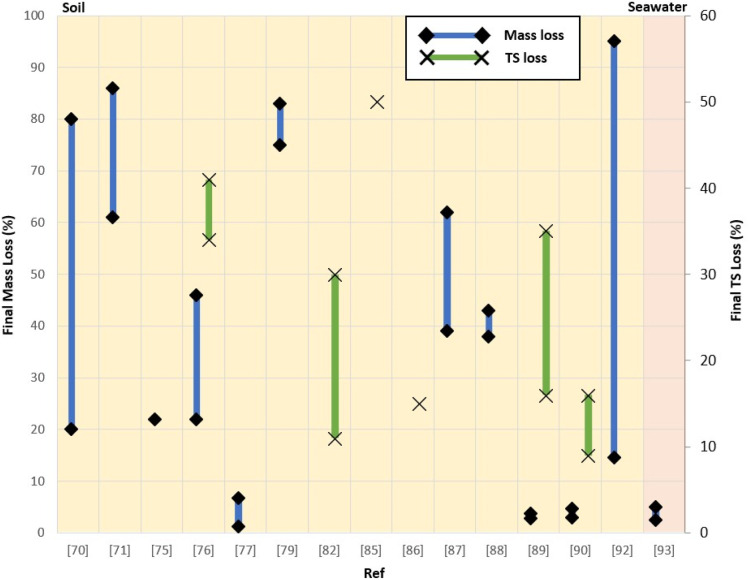
Results of current research on biodegradability of synthetic rubber, expressed as final mass loss and TS loss.^70,71,75–77,79,82,85–90,92,93^

A summary of biodegradation of some of the abovementioned synthetic rubbers formulated with natural material is shown in Fig. 5. Note that the comparison provided is for visualization of the range of weight loss and TS loss that have been reported by various research groups and should not be used for direct comparison of biodegradability of the materials due to the potential differences in biodegradation experimental conditions and setups. Based on the summary, the weight loss of the materials is largely in the range of 5 to 90%, while TS loss is about 10 to 70% after varying periods of biodegradation in various media such as soil and seawater.

### 3.6 Bio-based thermoplastic/synthetic rubber blend

Synthetic rubber could be blended with thermoplastics to produce thermoplastic elastomers (TPE) which exhibit both thermoplastic and elastomeric properties. Thermoplastic properties in TPE enable it to be remolded, providing an advantage over traditional thermoset synthetic rubbers in terms of processability and recyclability. Moreover, some of these thermoplastics are polyesters such as polylactide (PLA) and polycaprolactone (PCL) that contain hydrolysable ester linkages and they exhibit good hydrolytic degradation properties. The incorporation of these biodegradable thermoplastics in conventional synthetic rubber could introduce biodegradability in an otherwise non-biodegradable petroleum-derived synthetic rubber. PLA is a biobased polymer that is commonly produced from corn or sugar beet, and it has been widely used in the development of green plastics, 3D printing filaments, and as a component of polymer blends. Superior mechanical strength of thermoplastic vulcanizates (TPV) produced from blending of polylactide (PLA) with ethylene-*co*-vinyl acetate (EVA) was reported by Ma *et al.* in 2015. 2,5-Dimethyl-2,5-di(*tert*-butylperoxy)hexane (AD) was added to the blend as a radical crosslinker to generate interpenetrated network structure of PLA and EVA.^100^ At 1 wt% of AD, PLA/EVA blend of ratio 40 : 60 exhibited considerably higher TS (21 MPa) than the same ratio blend without AD (8 MPa). However, the EB (210%) was reduced to one-third of the non-crosslinked blend (600%) with the addition of the dynamic crosslinks, but the value was still considerably higher than the petroleum-based thermoplastic blend of PP and EPDM, at which the EB was merely 120%. The reduced tensile set and increased hardness with the AD dynamic crosslink are beneficial for the development of tough and flexible thermoplastic elastomer. The TPV experienced 5% weight loss after being immersed in aqueous alkali for 10 weeks, and from FTIR analysis, absorbance peaks from the PLA components in the blends were no longer visible in the degraded TPV samples. This confirms the partial degradability of the TPV and indicates the degradation proceeded in the PLA phase rather than the blend as a whole.

PCL is a polyester that is an important material in many medical applications such as surgical implants, bone tissue engineering or drug delivery due to its excellent biocompatibility, high biodegradation rate and mechanical strength. It is noteworthy that production of PCL is achieved through the ring-opening polymerization of ε-caprolactone or polycondensation of 6-hydroxyhexanoic acid, both monomers are usually derived from fossil fuel.^101^ Recently, Pyo *et al.* reported the bio-based route for the synthesis of the monomers of PCL through a series of microbial–chemical reactions of biomass, particularly fructose.^102^ This adds PCL to the list of originally fossil fuel derived polymers with bio-based available monomers, and subsequently improved the sustainability of the material. Tran *et al.* reported a fully degradable polymer semiconductor with elastomeric behaviour for application in transient electronics, especially skin-inspired electronics.^103^ Such polymer was obtained by blending polycaprolactone-based polyurethane (E-PCL) which serves as the elastomer matrix, with a polymeric semiconductor. Aldehyde-functionalized diketopyrrolopyrrole (DPP) was polymerized with *p*-phenyldiamine into a polymer semiconductor containing imine bonded backbone. Resultant biocompatible blend exhibit extremely high EB of more than 1000% with high TS and minor hysteresis at 50% strain for more than 10 cycles. The semiconductor was reported to be able to self-assemble and form nanoconfined fibril aggregates in the elastomer matrix, deriving its ability to depress crystallization and crack formation under applied strain, and consequently produce electrical properties that are stable under strained condition. Imine bonds in the semiconductor can be degraded with acid into monomers, while PCL is known to be hydrolytically degradable. As a result, the film of the blend had about 50% weight loss within 45 days when immersed in an acidic solution of trifluoroacetic acid, proving the degradability of the said blend. The ability of PCL to be degraded in various environments makes it a good candidate for degradable polymer use.

Thermoplastic poly(ester urethane) (TPEU) produced from PCL, hydrogenated 4,4′-methylene diphenyl diisocyanate (MDI), and different lengths chain extender (2-ethylurea diol derived from amino acid) was reported by Brannigan *et al.*^104^ The longer length of the chain extender makes the TPEU more hydrophobic and lowers the water uptake capabilities. This resulted in a change in the hydrolytic degradation mechanism shifting from bulk to surface erosion, slowing the overall degradation process. Fortunately, the degradability is not severely affected and all investigated TPEU fully degraded within 100 days in aqueous alkali. In a separate study, Gregory and William improved the alkaline hydrolytic degradation of triblock polyester consisting of blocks of poly(phthalic anhydride-*co*-4-vinylcyclohexene oxide) and PCL by forming ionomers through reaction with a base such as hydroxide of sodium or lithium.^105^ Carboxylation of the polyester through thiol-ene reaction was initiated by UV between vinyl pendant group of 4-vinylcyclohexene oxide and 3-mercaptopropionic acid. The formation of ionic bonds strengthens the mechanical properties, resulting in high TS, EB and elastic recovery, making the polyester tough, elastic and resilient. Additionally, it was more capable of absorbing water and degrading through alkaline hydrolysis. 75% of sodium–carboxyl group neutralized sample undergoes complete mass loss in 10 hours, while non-neutralized sample shown up to 90% mass loss in 25 hours, and the non-carboxylated samples were not degraded throughout the test period. The thermoplastic behaviour of the ionic polyester was confirmed with high retention of TS and EB after 3 cycles of the hot pressed (200 °C, 20 MPa). The facile modification process enables the properties to tune over a wide range with a straightforward method.

Poly(glycerol sebacate) (PGS) is a polyester that is used as a biomaterial in a wide range of medical applications such as tissue engineering, scaffolds and cell culture systems due to its excellent biocompatibility and unique properties.^106^ Recently, the development of a 3D printable porous elastomeric scaffold of PGS-acrylate has been reported.^107^ The thermal curing post scaffold fabrication (post-cure) can further enhance and tune the mechanical properties of the scaffold, and the process also affects the scaffold's degradability. When the post-cure duration was little or none, the rate of alkaline hydrolytic degradation of the scaffold was higher than those of PGS, and the rate decreased with the increase in the post-cure duration. The aqueous degradation process of poly-(butylene terephthalate)-*co*-(tetramethylene ether)glycol terephthalate (PBT-PTMG) was reported by Diaz *et al.* using accelerated degradation in distilled water at elevated temperature.^108^ They found that the degradation process followed a temperature dependant pseudo zero order kinetic, and the mechanical behaviour change during degradation is related to the degradation-induced crystallinity change. Basically, the formation of the crystalline region following the rearrangement of low molecular weight chains scissored by ester bond hydrolysis during degradation increased the storage modulus and shifted the mechanical behaviour of the material from elastomer to brittle after 3 days.

In 2015, Hu *et al.* reported a novel bio-based polyester (PLBSI) synthesized through polycondensation of lactic acid, sebacic acid, itaconic acid and 1,4-butanediol.^109^ Varying the amount of lactic acid in the formulation changed the mechanical behaviour of the resultant material. Reduction in compositional regularity caused by the methyl group side chain from the incorporated lactic acid allows the copolymer to transit from plastic to elastomer. Another linear polyester (PBPSSI) was synthesized by Hu *et al.* in 2016, involving five bio-based monomers, *i.e.* 1,3-propanediol, 2,3-butanediol, succinic acid, sebacic acid and itaconic acid.^110^ The resulting bioelastomer exhibited excellent thermal stability and good biocompatibility. In both PLBSI and PBPSSI, the TS was largely improved when the bioelastomer was reinforced with silica, while EB was not significantly affected compared to the unreinforced counterpart. Both composites have mechanical properties in the acceptable range for many engineering applications. However, the *in vitro* degradability in phosphate buffer saline was largely inhibited when both bioelastomers were peroxide-crosslinked, with about 35% mass loss in 30 days in unreinforced bioelastomers, to negligible degradation in the crosslinked composites. According to the authors, PBPSSI exhibited high degradability in suitable environment and have properties that could be suitable for a wide range of engineering applications. As for PLBSI, the similar lactate group as in PLA allows both polymers to be highly compatible with each other, and the resultant blend exhibited good toughening effect, improved EB and impact strength by 30 and 15 times, respectively compared to neat PLA.

**Fig. 6:**
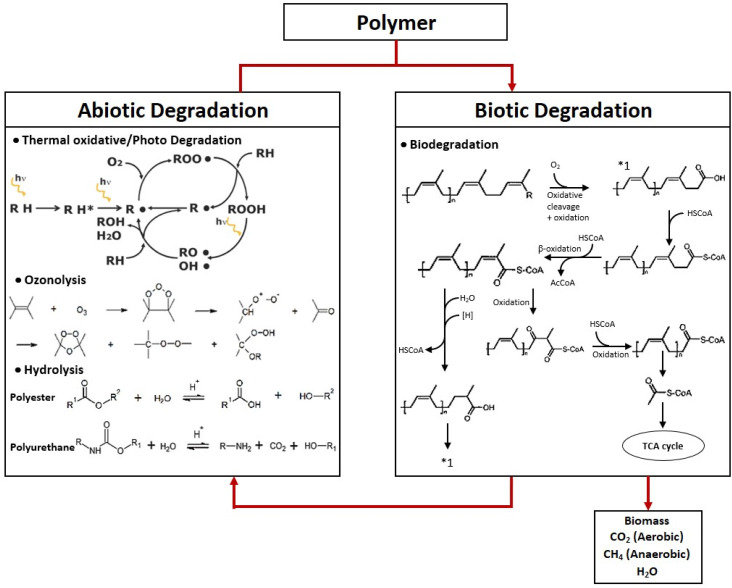
Mechanisms of various environmental degradation of polymeric materials.^114–116^

Degradation process of polymeric materials could be categorized into abiotic and biotic degradation. Biotic degradation is also known as biodegradation, where the degradation process is contributed by the activity of microbes, while abiotic degradation refers to the degradation of polymers triggered by physiochemical conditions, such as photolytic degradation by light or hydrolytic degradation in the presence of water.^111–113^ Mechanisms of various types of environmental degradation are summarized in Fig. 6. Often, the overall degradation process is not limited to one specific mechanism only. For example, oxo-biodegradation often begins with abiotic oxidative degradation followed by biodegradation.^112^ Oxidative degradation is often triggered by heat or light in the presence of oxygen, causing polymer chain oxidation and chain scission. Consequently, fragmented and lower molecular weight products containing higher concentration of oxygen-carrying groups such as carbonyls and hydroxyls are produced, and this has been reported to facilitate the subsequent biotic degradation.^113^ Although most of the biobased plastic/synthetic rubber blends summarized here reported improved abiotic degradability of the product, there is a lack of report on the microbial degradability of the rubber blend. Nevertheless, the improvement in the abiotic degradability of the blend is expected to be beneficial to the overall degradability.

## 4.0 Improving sustainability and properties of synthetic rubbers with biologically sourced materials

### 4.1 Bio-based fillers in synthetic rubbers

**Fig. 7:**
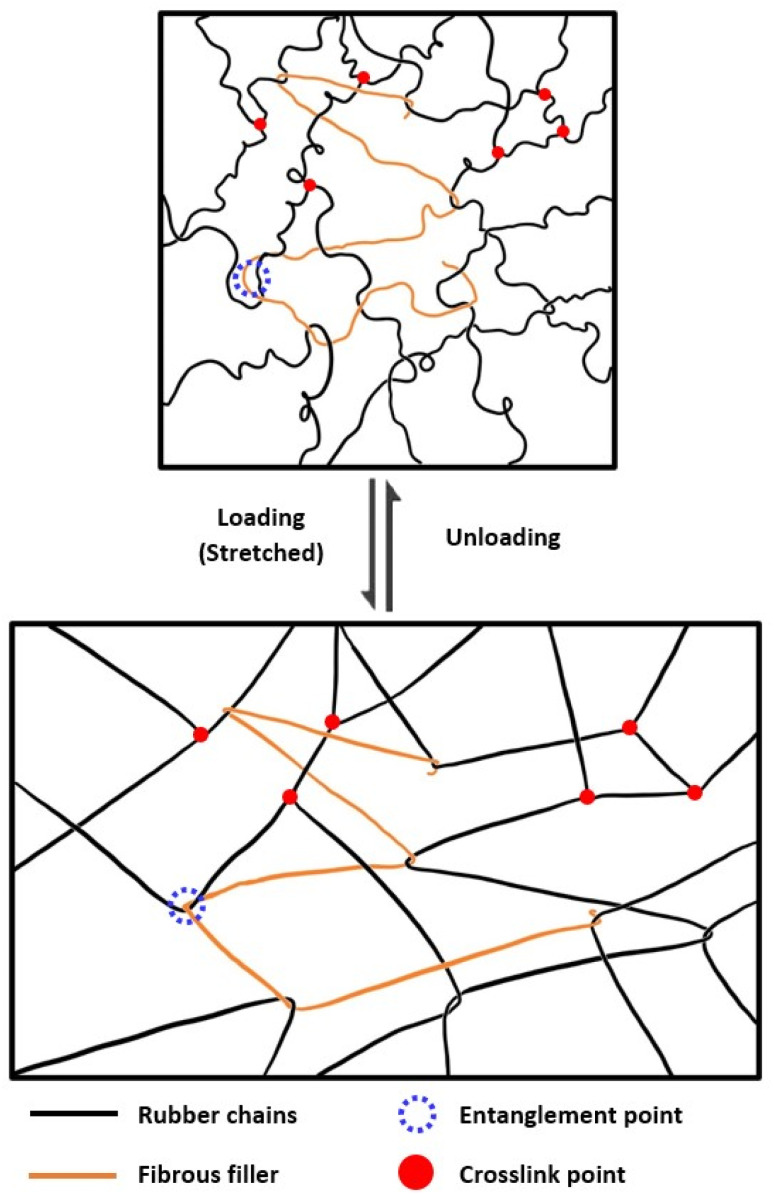
Mechanical strength reinforcement by fibrous fillers through physical entanglement.^117^

Much research had been conducted recently to incorporate renewable materials into the formulation of synthetic rubber to improve their performance and sustainability. Starch is one example of such material and it has been used as green filler in synthetic rubber formulations due to its low cost, high abundance, and excellent biodegradability. Generally, the introduction of filler in a rubber could improve its mechanical performance and one of the reinforcing mechanisms is through the physical entanglement that took place between the fillers and the rubber chains, illustrated in Fig. 7. Corn starch powder used as filler in SBR compounding showed higher TS, EB, and tear strength with increasing corn starch concentration. The cure characteristic such as cure rate and torque also improved compared to the unfilled form. However, the mechanical properties of reinforcement are still inferior largely when compared to silica, as the interfacial adhesion between unmodified starch with petroleum-based rubber matrix is low.^73^ Modification of starch granules is often carried out to improve this interaction for better compatibility and greater properties enhancement. Sago starch oxidized (OSt) with hydrogen peroxide was investigated for its role as filler in a sulphur-free compounding system.^118^ The high carboxyl group content (57%) in OSt improved the TS and EB by 304% and 199%, respectively and this improvement was attributed to the formation of ionic crosslink with zinc oxide. The filled elastomers also exhibited improved antibacterial ability with evident inhibition of bacterial growth at low pH. However, this could also imply lower biodegradability of the composite so the selection of materials and the modification technique should depend on the requirement of these properties.

Modified sago starch through acidic hydrolysis of sago starch solution at elevated temperature results in lower crosslink density in XNBR, and the rubber composite was reported to have poor mechanical properties.^75,119^ It was found that low molecular weight dextrin had low interfacial adhesion with rubber matrix and do not participate in crosslink, but the dextrin filler is expected to improve the biodegradability of the filled rubber because of the ease of consumption of dextrin by microbes. However, no result on biodegradation was presented in the study.^119^ The use of yeast fermented corn starch (MST) in SBR reduced mechanical properties due to weak interaction between filler and rubber matrix.^120^ The increase in the concentration of MST up to 100 phr improved the cure rate and crosslink density of the composite as the hydroxyl group take part in crosslinking and MST acts as an activator for vulcanization accelerator. Excellent water absorption and water swelling capability suggested its advantage in agriculture such as controlled fertilizer release and water retention.

The effect of using five different coupling agents separately in SBR filled with starch-*g*-PMMA was reported by Li *et al.* The coupling agents used in the study include styrene-*co*-maleic anhydride (SMA), bis[3-(triethoxysilyl)propyl]tetrasulfide (TESPT), 3-aminopropyl triethoxysilane (APTES), 3-mercaptopropyl triethoxysilane (MPTES) and MDI.^121,122^ Generally, these coupling agents slightly improved the TS, but produced different effects on EB. Among them, MDI produced the most significant influence on the EB and two possibilities that could have led to the improvement in the filler-matrix adhesion were proposed, *i.e.*, the formation of urethane linkage with starch, and the interaction of aromatic benzene rings between MDI and styrene moieties. MDI coupled composite also exhibits improvement in the initial thermal degradation temperature and thus the thermal stability of the composite. The study of different content of SMA showed improved TS and negligible EB change with increasing SMA content in the starch-*g*-PMMA/SBR composite up to 5 phr. Urethane polyfunctional monomer based on sucrose molecules was used as a co-agent in electron beam irradiation curing of EPDM rubber.^123^ Sucrose was diurethane-bonded with hydroxyethyl methacrylate and MDI. At 5 phr loading of the polyfunctional monomer, the crosslink density of the rubber composite improved, resulting in greater swelling resistance and higher thermal stability. A controlled antibacterial material release was incorporated in PDMS/starch blend through a complex formed by povidone-iodine (PVPI) and starch granule fillers.^124^ Sustained antibacterial activity for more than 1 month can be achieved through controlled release of iodine with the formation of iodine–starch complex when the composite is in contact with water.

The use of microcrystalline cellulose (MCC) as a multifunctional material in rubber compounding had been reported by various groups. In the SBR/BR blend, MCC act as a processing aid and reinforcing filler.^125^ Although the abrasion and wear resistance were slightly reduced when silica filler was replaced with MCC, the improvement in dry grip, heat build-up resistance, and processability make the composites' overall mechanical properties fall within the acceptable range for tire tread application. In 2018, Akbay and Güngör reported the extraction of cellulose from waste banana peels through sequential boiling in water and vinegar, followed by drying and grinding. Based on their report, incorporation of the cellulose in EPDM has resulted in better cure characteristics of the rubber composite.^126^

Acid hydrolysis of MCC produces cellulose nanocrystals or more commonly known as nanocrystalline cellulose (NCC). NCC has a smaller particle size with a higher surface area to volume ratio. The concentration of acid used for MCC hydrolysis greatly affects the yield and filler performance. The optimum concentration of 5 M HCl was reported to produce the smallest particle size while retaining a high yield. The use of excessively high acid concentration should be avoided as it could favour depolymerization of the cellulose and reduce them into amorphous sugar molecules.^127^ Compared to MCC, NCC was reported to have better interfacial adhesion, compatibility, and dispersion in both NBR and XSBR.^127–129^ The mechanical reinforcement trend of NCC is similar to that of MCC, in that TS, tear strength, and hardness increased with increasing filler content, whereas EB increased only slightly at a certain filler concentration before decreasing at higher filler loading. The inverse reinforcement between TS and EB was also seen in most conventional synthetic rubber fillers such as carbon black, silica, and clay materials. Cellulose agglomerates easily in the rubber matrix because cellulose contains a high concentration of hydroxyl groups, which is becoming more common when the matrix–filler interaction is weak. By ensuring the optimum concentration of filler in the rubber, a percolated network of NCC was achieved in the SBR. Consequently, the rubber exhibited improved tear strength and this was attributed to the higher force required to break the percolated filler network for crack propagation.^130^ The use of NCC with zinc oxide as fillers formed Zn–cellulose through the formation of ionic bonds, improving the dispersion of both fillers in the NBR matrix and resulting in a composite with superior mechanical properties than both singly filled-NBR.^131^ Other recent works involving modification of NCC filler for synthetic rubber is summarized in Table 2.

**Table 2:** Surface functionalization of NCC fillers for synthetic rubber

Rubber matrix	NCC modification	Result	Ref.
XNBR	Oxidation with hydrogen peroxide	Denser and stronger crosslink network due to extra carboxyl group for crosslink	132
NBR	Acetylation with acetic anhydride	More compatibility and good dispersion with increased hydrophobicity	133
CR	Graft polymerization with PLA	Good compatibility and dispersion from hydrophobic and good water dispersibility	134
NBR	Thiol-functionalized with silane	High TS (+375%) and EB (+116%) with 0.98% hydroxyl group substitution	135
XSBR	Carboxylation with citric acid	5-Fold TS, dual crosslink network with poly(ethylene glycol) diglycidyl ether	136

The potential of 2,2,6,6-tetramethylpiperidine-1-oxyl (TEMPO) oxidized cellulose nanofibers (TCNF) to produce a blend with EPDM was reported by Kucuk *et al.* in 2020 with the aim to reduce petroleum-based materials in the rubber composite.^137^ The findings suggest that a stronger composite with excellent weathering resistance targeted for automotive sealing applications can be obtained aside from reducing fossil fuel-based material in EPDM rubber. Superior TS vitrimer elastomer based on XNBR could be obtained with the addition of epoxidized soybean oil (ESO).^138^ Reversible dynamic β-hydroxyl ester bonds formed between the epoxy group of ESO and carboxyl group of XNBR and TCNF gave the composite high mechanical strength retention after recycling and good shape memory ability through thermal treatment.

Lignin-based fillers are often used with other chemicals such as hybrid fillers or compatibilizers. Cationic lignin obtained through the reaction of lignin fibrils with glycidyltrimethylammonium chloride, when in use in binary filler systems with MMT formed a lamina of MMT with cationic lignin anchor on two sides of the nanosheet.^139^ This hybrid filler enhanced the mechanical properties of SBR, improving TS and EB by 110.7% and 39.1% compared to the unmodified lignin-filled SBR of the same filler content. Yu *et al.* reported the use of lignin to form an interpenetrating network with commercial epoxy resin in NBR to strengthen the rubber composite and improved its oil resistance.^140^ A thermoplastic composite could be achieved with the addition of GO filler in melt reactive mixing of NBR/lignin (ratio = 1 : 1) blend. The hydrophilic and high surface area GO platelet facilitated the dispersion of lignin fillers in the NBR to produce high TS and moderate EB composite with thermoplastic characteristics.^141^ The effect of functionalization of lignin and its performance in SBR composite was reported by Jiang *et al.* in 2018. Unmodified lignin, aldehyde-functionalized lignin, and hydroxylpropyl-functionalized lignin exhibit random aggregate, spherical granule, and supramolecular domains, respectively. The difference in the aqueous alkali aggregation structure made the filler dispersibility in the rubber matrix in the increasing order of unmodified < aldehyde < hydroxylpropyl.^142^ ENR compatibilizer added in these composites managed to improve the dispersibility of these fillers. Specifically, hydroxylpropyl lignin-filled SBR with ENR compatibilizer had improved mechanical properties compared to the non-compatibilized counterparts, at the same time having a better wet skid resistance and low rolling loss than other samples. The result reflects the potential of hydroxylpropyl lignin filler for the development of green tires.

### 4.2 Bio-based processing aid or plasticizer for synthetic rubber

Aromatic oils derived from petroleum are often used in rubber processing to increase the processability of the rubber compounds through the action of plasticizing, reducing the viscosity, improving the processability, and enabling better filler incorporation without significantly affecting the final properties of rubber formed. The use of plant oils as rubber processing aids is getting attention because petroleum processing oils are generally toxic and carcinogenic.^143^ Epoxidized palm oil (EPO) could act as a softener and plasticizer to increase the free volume, reduce the density, and subsequently improve rubber chain mobility.^144,145^ Mechanical properties of SBR incorporated with EPO improved when the EPO content was below 35 phr. Below this concentration, the mechanical strength and elasticity of the composite increased with increasing EPO content.^146^ The addition of EPO processing oil reduced the viscosity of the compounded latex, and the crosslink density, as well as mechanical strength of the resultant composite films, was enhanced.

Soybean oil also can be used in plasticizing ground tyre rubber (GTR) to produce highly reclaimed rubber which can then be reused as a rubber matrix substitute.^147^ When the GTR/soybean oil mixture is used in the sulphur-cured system, the mixture functions as a reactive plasticizer by facilitating the recombination of broken rubber chains and providing double bonds for sulphur crosslink. Besides, the core–shell structure of carbon black bound by rubber from reclaimed rubber also helps in the reinforcement of mechanical strength. 20 phr substitution of SBR with the reactive plasticizer improved TS by 30% and could provide a new approach to utilizing waste materials, creating added value products. Hydrolysis of soybean oil in alkaline conditions resulted in soybean fatty acid that has a similar plasticizing effect in NBR composite.^148^ The flowability of the latex improved with reduced Mooney viscosity and resulted in a longer cure time. Incorporation of 4 phr of soybean oil fatty acid resulted in maximum crosslink density (maximum TS with good EB) and higher thermal stability, with the TS of the NBR composite reported to be higher than that incorporated with petroleum oil derivative, dioctyl phthalate (DOP).

Castor oil consisting of mainly ricinoleic acid and jatropha oil with high unsaturated fatty acid content has been reported as substitute processing aid oils in SBR composite.^149^ The cure characteristics of the rubber composite improved with the increase in the oil concentration while TS remain unaffected. The fact that these oils are inedible makes them even more attractive as potential replacements to the fossil-based aromatic processing oil. Cardanol is a class of lipid consisting of a phenol group with usually a long alkyl side chain and is the main constituent in cashew nut shell liquid. XNBR grafted with cardanol had been reported recently by Samantarai *et al.* in 2019.^150^ The latex had lower Mooney viscosity, and a higher plasticity retention index (PRI), indicating the plasticizing ability of cardanol. Grafting of cardanol through the oligomerized phosphorylated cardanol in NBR reported similar behaviour in the latex, replacing the need for DOP aromatic processing oil.^151^ Both composites that incorporated cardanol-based plasticizer exhibit low *T*_g_, high thermal stability, and are biocompatible.

### 4.3 Bio-based materials that require no extraction or isolation procedure

Recently there are more reports on the use of materials from biological sources that were obtained without extraction or isolation process. This greatly simplifies the material preparation process, making it easier and more practicable to be developed into industrial large-scale manufacturing and involves the usage of lesser chemicals. For example, in recent years, the use of cellulosic materials obtained directly from sources without extraction is gaining attention to evaluate the necessity of pure cellulose extraction.^152–157^ The process of cellulose isolation usually requires a vast amount of chemicals and produces many wastes, and this could be deemed as an obstacle in achieving the sustainability goal. Recent reports of other naturally sourced materials that were obtained without extraction or isolation are tabulated in Table 3. These materials consisted of a mixture of materials that could provide synergistic effects in property reinforcement. However, these effects were not investigated thoroughly as the complete compositional analysis of these materials was not carried out in most of the research. One of the challenges in using these materials on the industrial scale also comes from the compositional aspect. The composition of each constituent in these materials ranged widely as they are products of biological systems, and the composition is affected by many factors such as weather, climate, environment, lifetime, and others. The reinforcement ability could change drastically with little change in the composition of the material. On the other hand, component extraction from the materials provides an easier solution to control the quality of the said materials.

**Table 3:** List of research involving biologically sourced materials without extraction or isolation

Material	Treatment	Size	Rubber matrix	Result	Ref.
Blue crab shell (*Callinectes sapidus*)	Washed, dried & grinded	<250 μm	EPDM	Improved cure characteristic – act as vulcanization activator, improved crosslink density & thermal stability	152
Coconut shell powder	Cut & pulverized	150–180 μm	NBR	Mechanical properties reinforcement	153
	Washed, dried & grinded	<240 μm	NBR/SBR	Hybrid filler crysnanoclay, improved hardness	154
Cereal straw	Cut & grinded	N/A	EPDM, PDMS, SBR, NBR	General: shortened cure time	155
				PDMS & EPDM: high aging resistance	
				NBR: TS & tear strength improved	
Chicken feather	Washed, barbules removed & grinded	N/A	SBR	Improved stiffness	156
Chicken eggshell	Terpolymer grafted	570–690 μm	NBR	Good substitute to CaCO_3_	157
Flax fiber	N/A	N/A	EPDM	Electron beam irradiation cured, increased water uptake	158
Milk	Casein precipitation & grinded	<250 μm	EPDM	Better cure rate, higher crosslink density & EB	159
Olive solid waste	Size separated & dried	≤45 μm	XNBR	Improved interfacial interaction & TS	160
Peanut shell powder	Silane treated	567 nm	SBR	Higher stiffness, faster cure rate	161
Plant gum (*Spondias dulcis*)	Precipitated & dried	N/A	CR	Increased limiting oxygen index & flame retarding property	162
Rice husk	Dried & grinded	150 nm	SBR	Improved thermal stability, flame retardancy, mechanical strength and antibacterial behavior	163
Green molokhia leaf	Dried, grinded and extracted	N/A			
Waste walnut shell	Silane treated	<250 μm & <500 μm	EPDM	Comparable mechanical properties with reduced filler amount, reduce use of petroleum material	164
Wood waste	Hydrolysis & silane treated	N/A	EPDM	Decreased water uptake, improved thermal stability, filler dispersibility and TS	165
	Grinded & potassium oleated treated	N/A	CR	Not effective substitute of silica	166
	Grinded	<1 mm	EPDM	Slight improved abrasion and tear strength, inferior mechanical properties	167
	Grinded	<250 μm	EPDM	Increased water uptake, crosslink density, TS, hardness with electron beam irradiation	168
Wool fiber	Cleaned & cut	N/A	SBR	Sharp decrease in mechanical properties (high loading)	169

The cost-effectiveness of naturally sourced materials will be more established if the acceptable levels of reinforcement in rubber properties could be achieved with minimal treatment of raw materials. Some of the reported examples include coconut shell powder, coir fibre and flax fibres.^153,158,170^ Coconut shell powder is an efficient replacement for the fast extrusion furnace (FEF) filler in NBR, resulting in higher TS. It also contributed to increased water uptake capabilities in NBR/SBR blend, a property which is desirable in improving the biodegradability.^154^ Coir fibre acts as a nucleating agent for crystallization of polypropylene (PP) in EPDM-*g*-methyl methacrylate/PP blend, and as physical entanglement compatibilizer between EPDM and PP for high mechanical strength and toughness. Flax fibre has good dispersion in EPDM through melt blending and the resultant material has good thermal stability, comparable to that produced with the petroleum counterpart. Silane modification is commonly performed on fillers to enhance the mechanical properties reinforcement of the filled material, and such modification enabled a wider range of renewable raw materials to be used as filler. Peanut shell powder, waste walnut shell and wood fibre fillers with silane modification resulted in better cure characteristics in SBR, comparable mechanical and thermal properties with largely reduced filler content in EPDM composite, and enhanced stiffness in EPDM, respectively.^161,164,165^

## 5.0 Future prospect & conclusion

Currently, the supply chain of natural rubber is at risk with sole rubber tree species *Hevea brasiliensis* supplying the market predominantly, and the bulk of the rubber plantations are constricted in the Southeast Asia region. A disease wipe could easily cause a serious shortage of natural rubber supply, rendering the search for alternatives necessary. Fortunately, with the help of large rubber manufacturing companies, the commercialization of natural rubber alternatives such as those from Guayule and Russian dandelion has been realized. Although the volume of such rubber is significantly smaller, it is increasing steadily at a slow pace and could possibly serve as a backup supply in times of emergency. Natural rubber had been an excellent rubber material, but some applications require elastomeric materials with durability, chemical resistance, and/or thermal stability that exceed natural rubber. This puts the demand for synthetic rubber high and increases the rate of depletion of fossil fuel sources. Without any corrective action, the current reservoir is expected to be completely depleted in less than a century.

Fortunately, the synthetic rubber and plastic industries have started to replace some of the conventional raw materials in the product development with those derived from natural or biological sources. Although the percentage of this initiative is rather insignificant compared to the enormous production volume of fossil fuels-based counterparts, it had increased steadily over the past 10 years in the hope of at least delaying fossil fuel depletion. The rapid development in fermentation technology has resulted in new varieties of biologically derived raw materials with good cost-effectiveness and high production volume. The biologically derived raw materials are mostly used as fillers in synthetic rubber formulations. While modifications of fillers are inevitable because of the difference in surface chemistry with the rubber matrix that could result in poor compatibility and weak reinforcement, research should be carried out to simplify the process and minimize chemicals used and waste produced.

Another major problem is the non-biodegradability of synthetic rubber products which has led to environmental pollution and an increase in landfill areas. In some of the reported works, the biodegradability of synthetic rubbers were enhanced by incorporating biodegradable raw materials in the formulation, usually those which are renewable in nature. Some promising results were reported, with weight loss achieving as high as 90% and tensile strength loss up to 70% throughout the biodegradation process. It is however noteworthy that similar effect of enhanced biodegradation cannot be expected in every synthetic rubber simply by incorporating bio-based ingredients in the formulations. Factors such as modifications of these ingredients or reactions while in the rubber matrix could alter the chemical structure or reactivity, and subsequently limits the biodegradability. It is reasonable to expect that the consumption or digestibility of the material by microbes depends on the newly formed chemical structure, and not the original ones as they were extracted. Biodegradation tests are often not carried out in synthetic rubber research presumably because there is a lack of standard methods to effectively evaluate the biodegradation of rubber materials. This is evident when some recorded TS loss to quantitate the extent of biodegradation, while some reported weight loss. Furthermore, these tests were conducted under different conditions and environments, making it difficult to make any direct comparison. As sustainability is an important topic in synthetic rubber research and industry, it is necessary to come up with biodegradation test methods specifically for rubber so that the effect of those bio-based materials in synthetic rubber can be evaluated more accurately.

## Data availability

The datasets generated during and/or analysed during the current study are available from the corresponding author on reasonable request.

## Author contributions

Zhen Hern Boon: conceptualization, writing-original draft preparation, data curation. Yin Yin Teo: supervision, writing-reviewing and editing. Desmond Teck-Chye Ang: conceptualization, supervision, funding acquisition, writing-reviewing and editing.

## Conflicts of interest

There are no conflicts to declare.

## Supplementary Material
